# Supplementary figures and images for: Identification of a five-miRNA signature predicting survival in cutaneous melanoma cancer patients
Source: PeerJ. 2019 Oct 22;7:e7831. doi: 10.7717/peerj.7831 (PMC6814066; doi:10.7717/peerj.7831)

Volcano Plot

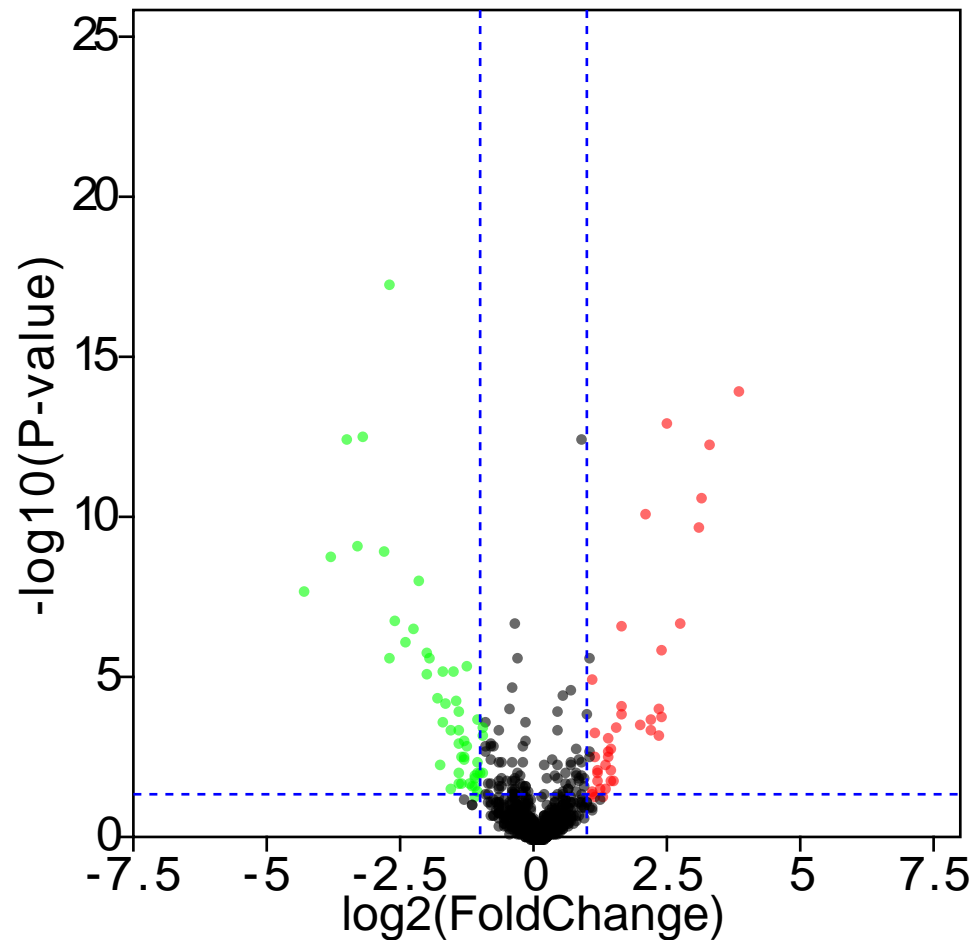

Supplement: Supplemental Information 1 — (A) The data in folder A is downloaded from GEO GSE35579 and used to screen for differentially expressed genes in melanoma and nevi. (B) The data in folder B was downloaded from TCGA to obtain miRNA expression profiles and clinical features of melanoma patients. (C) The data in folder C is used to demonstrate the process of determining the relationship between the expression of differentially expressed genes and the clinical features of melanoma patients. (D) The data in folder D shows how to determine the prognostic relationship between five differentially expressed miRNAs and melanoma patients by SPSS software. (E) The data in folder E is downloaded from DAVID, which is used to perform miRNAs target gene GO and KEGG analysis. [file peerj-07-7831-s001.zip › A_diff_PandM_vs_N-volGSE35579/volMandP-vs-naevus5.pdf]

## a five-miRNA signature

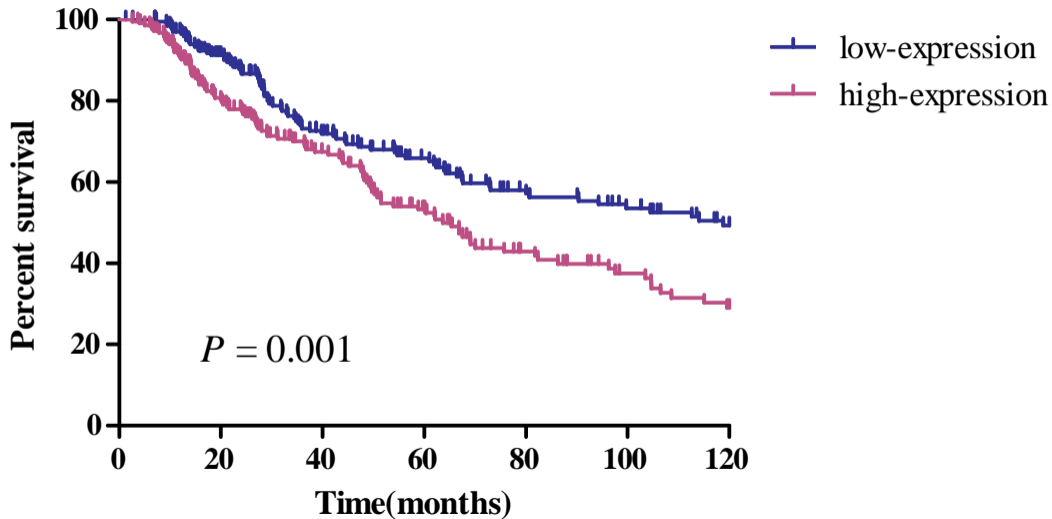

Supplement: Supplemental Information 1 — (A) The data in folder A is downloaded from GEO GSE35579 and used to screen for differentially expressed genes in melanoma and nevi. (B) The data in folder B was downloaded from TCGA to obtain miRNA expression profiles and clinical features of melanoma patients. (C) The data in folder C is used to demonstrate the process of determining the relationship between the expression of differentially expressed genes and the clinical features of melanoma patients. (D) The data in folder D shows how to determine the prognostic relationship between five differentially expressed miRNAs and melanoma patients by SPSS software. (E) The data in folder E is downloaded from DAVID, which is used to perform miRNAs target gene GO and KEGG analysis. [file peerj-07-7831-s001.zip › D_survival and Cox-regression/Survival results visualization with Prism 5/a five_miRNA signature-2.pdf]

# miR-204

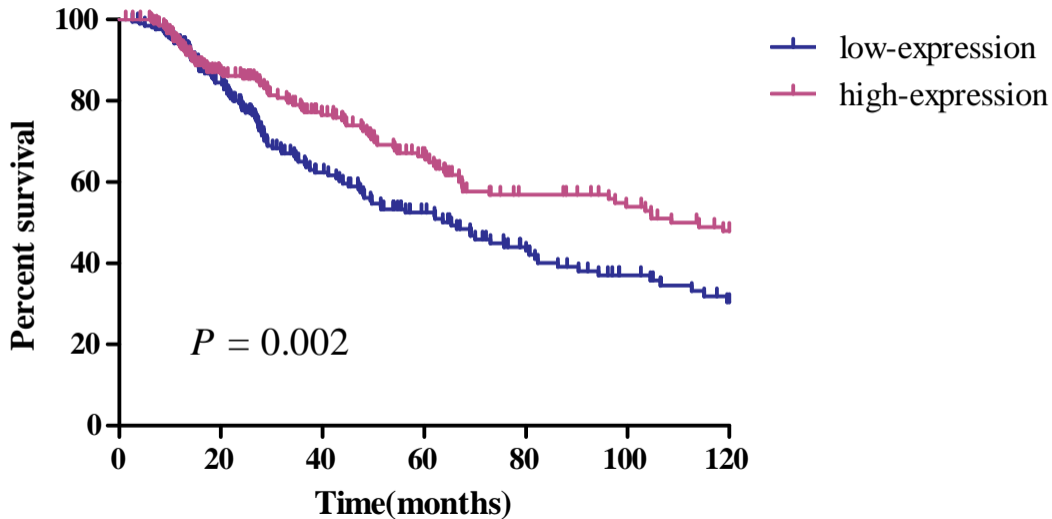

Supplement: Supplemental Information 1 — (A) The data in folder A is downloaded from GEO GSE35579 and used to screen for differentially expressed genes in melanoma and nevi. (B) The data in folder B was downloaded from TCGA to obtain miRNA expression profiles and clinical features of melanoma patients. (C) The data in folder C is used to demonstrate the process of determining the relationship between the expression of differentially expressed genes and the clinical features of melanoma patients. (D) The data in folder D shows how to determine the prognostic relationship between five differentially expressed miRNAs and melanoma patients by SPSS software. (E) The data in folder E is downloaded from DAVID, which is used to perform miRNAs target gene GO and KEGG analysis. [file peerj-07-7831-s001.zip › D_survival and Cox-regression/Survival results visualization with Prism 5/miR-204-2.pdf]

# miR-25

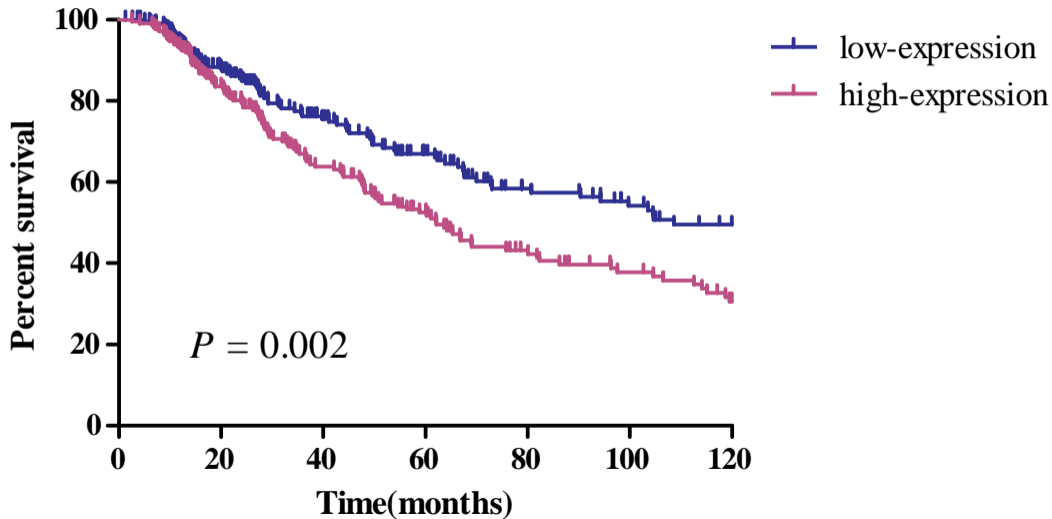

Supplement: Supplemental Information 1 — (A) The data in folder A is downloaded from GEO GSE35579 and used to screen for differentially expressed genes in melanoma and nevi. (B) The data in folder B was downloaded from TCGA to obtain miRNA expression profiles and clinical features of melanoma patients. (C) The data in folder C is used to demonstrate the process of determining the relationship between the expression of differentially expressed genes and the clinical features of melanoma patients. (D) The data in folder D shows how to determine the prognostic relationship between five differentially expressed miRNAs and melanoma patients by SPSS software. (E) The data in folder E is downloaded from DAVID, which is used to perform miRNAs target gene GO and KEGG analysis. [file peerj-07-7831-s001.zip › D_survival and Cox-regression/Survival results visualization with Prism 5/miR-25-2.pdf]

# miR-211

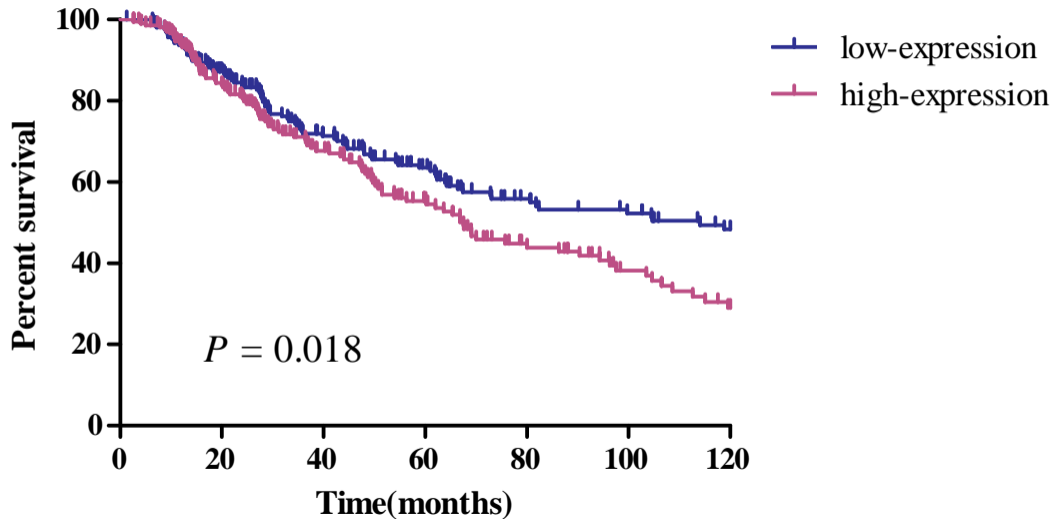

Supplement: Supplemental Information 1 — (A) The data in folder A is downloaded from GEO GSE35579 and used to screen for differentially expressed genes in melanoma and nevi. (B) The data in folder B was downloaded from TCGA to obtain miRNA expression profiles and clinical features of melanoma patients. (C) The data in folder C is used to demonstrate the process of determining the relationship between the expression of differentially expressed genes and the clinical features of melanoma patients. (D) The data in folder D shows how to determine the prognostic relationship between five differentially expressed miRNAs and melanoma patients by SPSS software. (E) The data in folder E is downloaded from DAVID, which is used to perform miRNAs target gene GO and KEGG analysis. [file peerj-07-7831-s001.zip › D_survival and Cox-regression/Survival results visualization with Prism 5/miR_211-2.pdf]

# miR-510

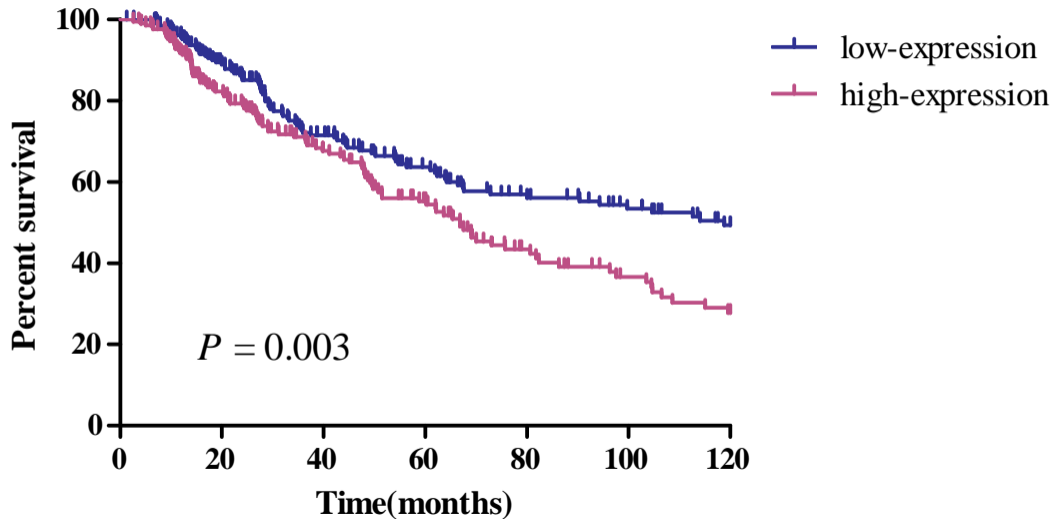

Supplement: Supplemental Information 1 — (A) The data in folder A is downloaded from GEO GSE35579 and used to screen for differentially expressed genes in melanoma and nevi. (B) The data in folder B was downloaded from TCGA to obtain miRNA expression profiles and clinical features of melanoma patients. (C) The data in folder C is used to demonstrate the process of determining the relationship between the expression of differentially expressed genes and the clinical features of melanoma patients. (D) The data in folder D shows how to determine the prognostic relationship between five differentially expressed miRNAs and melanoma patients by SPSS software. (E) The data in folder E is downloaded from DAVID, which is used to perform miRNAs target gene GO and KEGG analysis. [file peerj-07-7831-s001.zip › D_survival and Cox-regression/Survival results visualization with Prism 5/miR_510-2.pdf]

## miR-513c

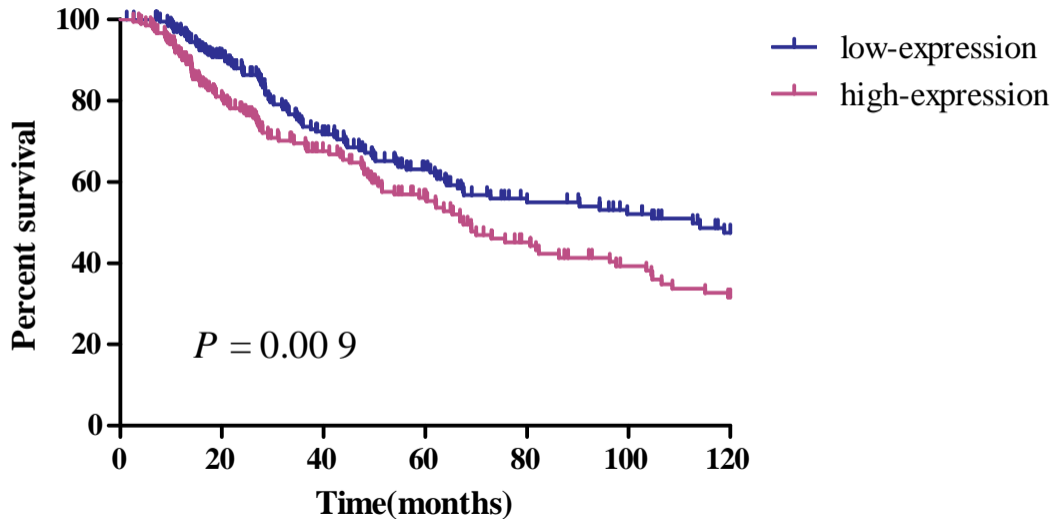

Supplement: Supplemental Information 1 — (A) The data in folder A is downloaded from GEO GSE35579 and used to screen for differentially expressed genes in melanoma and nevi. (B) The data in folder B was downloaded from TCGA to obtain miRNA expression profiles and clinical features of melanoma patients. (C) The data in folder C is used to demonstrate the process of determining the relationship between the expression of differentially expressed genes and the clinical features of melanoma patients. (D) The data in folder D shows how to determine the prognostic relationship between five differentially expressed miRNAs and melanoma patients by SPSS software. (E) The data in folder E is downloaded from DAVID, which is used to perform miRNAs target gene GO and KEGG analysis. [file peerj-07-7831-s001.zip › D_survival and Cox-regression/Survival results visualization with Prism 5/miR_513c-2.pdf]

A

miR-25

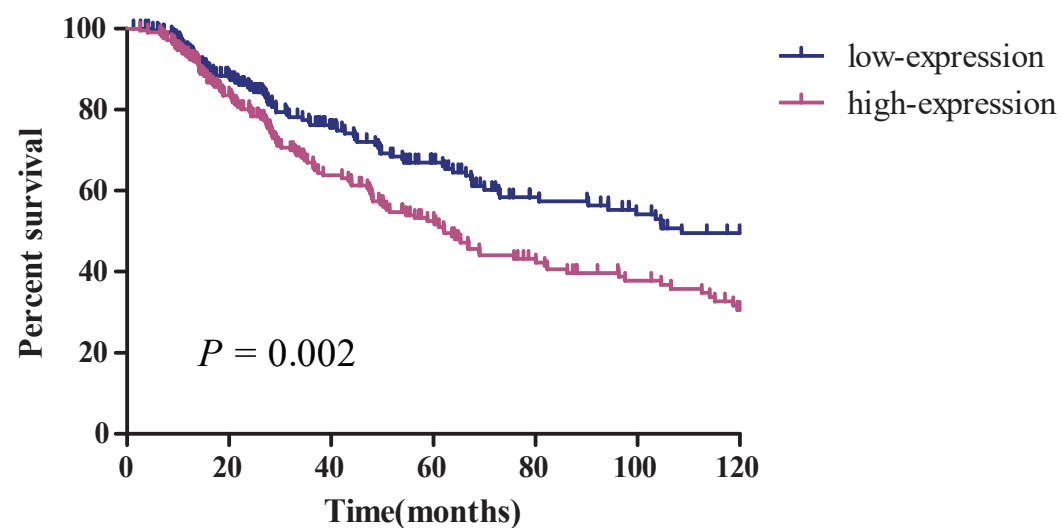

B

miR-204

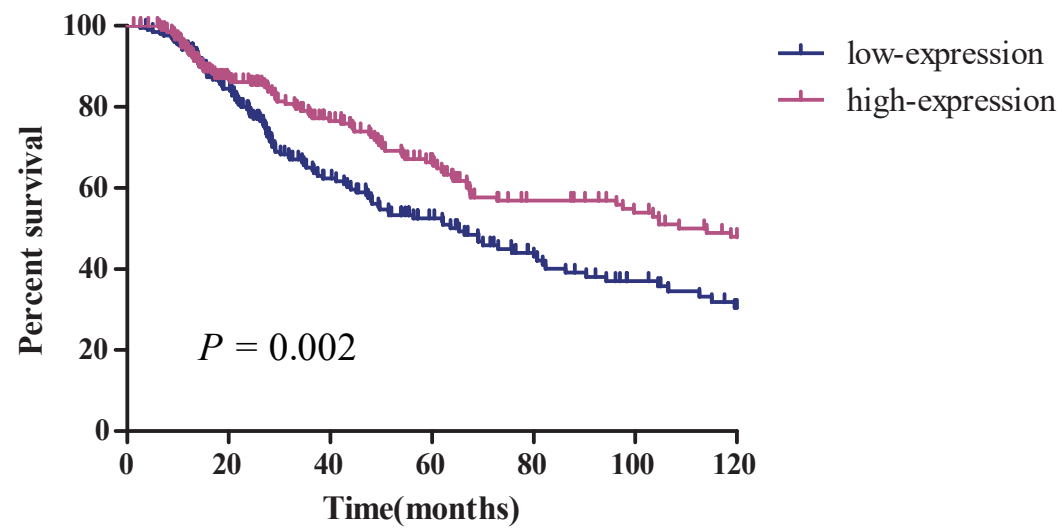

C

miR-211

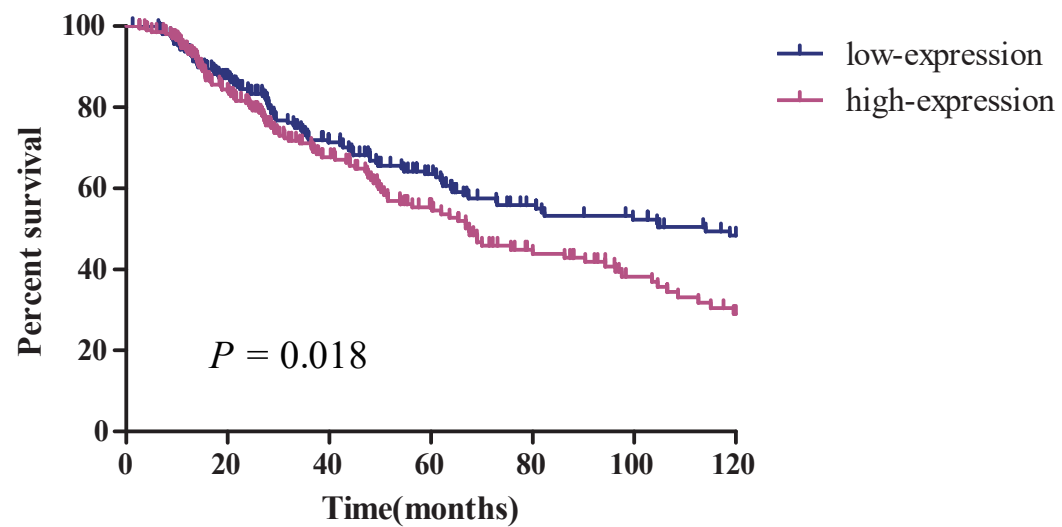

D

miR-510

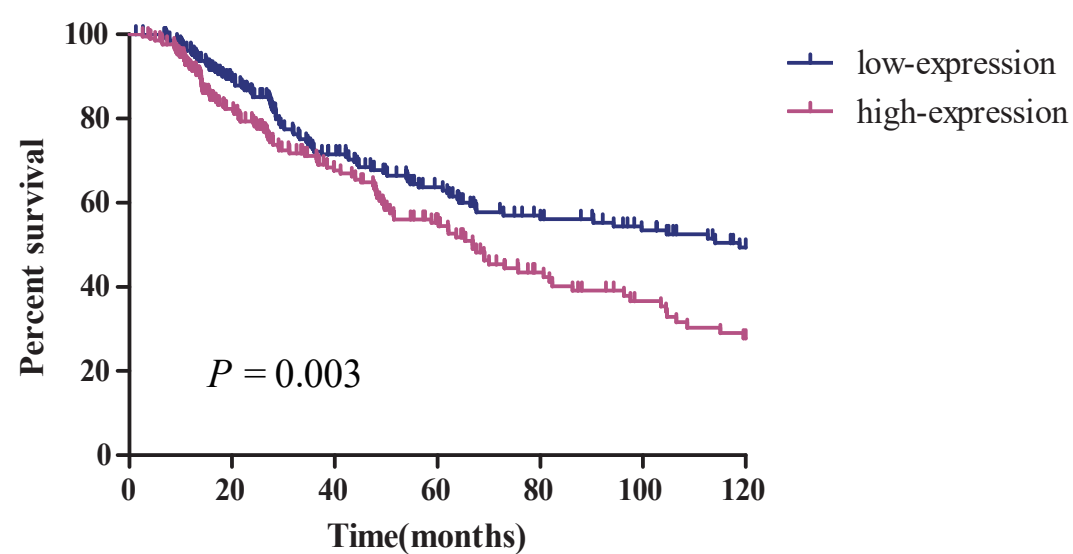

E

miR-513c

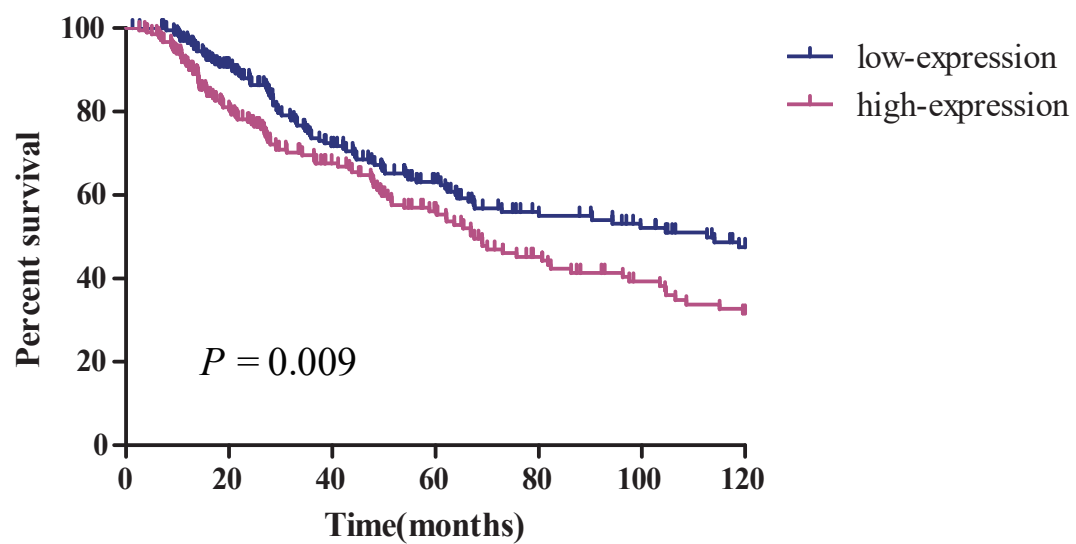

F

a five-miRNA signature

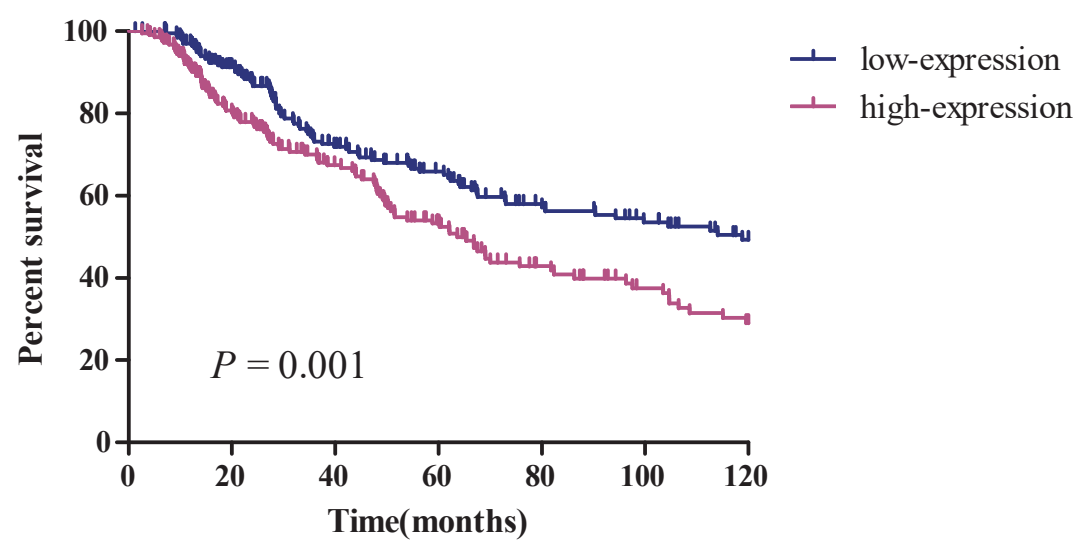

Supplement: Supplemental Information 1 — (A) The data in folder A is downloaded from GEO GSE35579 and used to screen for differentially expressed genes in melanoma and nevi. (B) The data in folder B was downloaded from TCGA to obtain miRNA expression profiles and clinical features of melanoma patients. (C) The data in folder C is used to demonstrate the process of determining the relationship between the expression of differentially expressed genes and the clinical features of melanoma patients. (D) The data in folder D shows how to determine the prognostic relationship between five differentially expressed miRNAs and melanoma patients by SPSS software. (E) The data in folder E is downloaded from DAVID, which is used to perform miRNAs target gene GO and KEGG analysis. [file peerj-07-7831-s001.zip › D_survival and Cox-regression/Survival results visualization with Prism 5/survival analysis.pdf]
